# DNA Damage Tolerance by Eukaryotic DNA Polymerase and Primase PrimPol

**DOI:** 10.3390/ijms18071584

**Published:** 2017-07-21

**Authors:** Elizaveta O. Boldinova, Paulina H. Wanrooij, Evgeniy S. Shilkin, Sjoerd Wanrooij, Alena V. Makarova

**Affiliations:** 1 Institute of Molecular Genetics of Russian Academy of Sciences, Kurchatov sq. 2, 123182 Moscow, Russia; lizaboldinova@yandex.ru (E.O.B.); shilkinevgeniy.chem@gmail.com (E.S.S.); 2 Department of Medical Biochemistry and Biophysics, Umeå University, 901 87 Umeå, Sweden; paulina.wanrooij@umu.se

**Keywords:** PrimPol, replication, DNA damage, mitochondria

## Abstract

PrimPol is a human deoxyribonucleic acid (DNA) polymerase that also possesses primase activity and is involved in DNA damage tolerance, the prevention of genome instability and mitochondrial DNA maintenance. In this review, we focus on recent advances in biochemical and crystallographic studies of PrimPol, as well as in identification of new protein-protein interaction partners. Furthermore, we discuss the possible functions of PrimPol in both the nucleus and the mitochondria.

## 1. Introduction

Human cells contain a variety of deoxyribonucleic acid (DNA) polymerases that differ in function and fidelity. Multisubunit replicative DNA polymerases Pol δ and Pol ε possess high fidelity and play a pivotal role in the replication of genomic DNA due to the stringent requirements of their active site [1], while Pol α, which forms a complex with primase, is responsible for the initiation of DNA replication during *de novo* ribonucleic acid (RNA)-primer synthesis at the origins of replication [2,3]. Pol γ is essential for mitochondrial DNA replication and repair [4,5,6].

In living organisms, however, DNA is subject to damage by various endogenous and exogenous chemical and physical factors such as reactive oxygen and nitrogen species, naturally occurring ultraviolet and ionizing radiation, and reactive chemicals from environmental, food and therapeutic sources [7,8,9,10,11]. The replication of damaged DNA (DNA translesion synthesis (TLS)) relies on specialized DNA polymerases, also called translesion DNA polymerases. Human translesion DNA polymerases include all members of the Y-family of DNA polymerases (Pol η, Pol ι, Pol κ, Rev1) as well as the B-family DNA polymerase Pol ζ [8,12,13]. They also include some A- and X-family DNA polymerases, such as Pol ν, Pol θ, Pol β, Pol λ, Pol μ [14,15,16,17]. Translesion DNA polymerases possess unique DNA damage bypass and fidelity profiles. Lesion bypass can be error-free or error-prone depending on the type of lesion and the particular translesion DNA polymerase that is involved in synthesis. Generally, translesion polymerases possess a wide and flexible active site and/or utilize non-canonical interactions during base-pairing and can therefore efficiently incorporate nucleotides opposite the site of damage. Because of the tolerance of the active site and the lack of 3′–5′ exonuclease activity, translesion DNA polymerases often demonstrate low accuracy of DNA synthesis; consequently, error-prone lesion bypass constitutes a leading mechanism of mutagenesis in eukaryotes.

In addition to DNA damage, non-B DNA structures and collisions between the replication and transcription machineries can also lead to replication fork stalling and cause replication stress and genome instability [18,19,20]. Our understanding of the processes and factors that help to resolve such collisions and the mechanisms of replication through natural DNA obstacles is still far from complete.

For a long time, a primase that forms a complex with Pol α was the only known eukaryotic primase. The Pol α-primase complex consists of the DNA polymerase catalytic subunit POLA1, the regulatory subunit POLA2 and the small catalytic and large regulatory primase subunits PriS (Prim1) and PriL (Prim2), respectively [21,22,23].

Prim1 belongs to the archaea-eukaryotic primase (AEP) superfamily. Many members of this superfamily possess both primase and DNA polymerase activities and play an essential role not only in initiation of DNA replication, but also undertake a wide variety of cellular roles in DNA replication, damage tolerance and repair, in addition to primer synthesis [24]. In 2005, Iyer L.M. et al. *in silico* predicted the existence of a new hypothetical single subunit human primase encoded by the gene *CCDC111* on chromosome 4q35.1 [25]. The protein encoded by *CCDC111* belongs to the NCLDV-herpesvirus clade of the AEP primases. In 2012 and 2013, this new enzyme was purified and characterized as a translesion DNA polymerase with low accuracy of DNA synthesis and primase activity. It was initially presented by the L. Blanco group at several meetings in 2012 and 2013 (Sevilla 2012, Banff 2013 and others). In 2013, three groups published research articles describing the new enzyme [26,27,28]. The protein shares the same active site for the DNA polymerase and primase activities and was named PrimPol (“Prim”—primase, “Pol”—polymerase).

Subcellular fractionation and immunodetection studies indicated that human PrimPol is present in both the nucleus and the mitochondrial matrix in human cells. In particular, in Hela cells PrimPol is distributed between the cytosol, mitochondria and nucleus with 47%, 34% and 19%, respectively, in each respective compartment [27]. With some exceptions, homologues of human PrimPol were found in many eukaryotic unicellular and multicellular organisms, including animals, plants, fungi and protists [27,29]. However, PrimPol-related proteins were not identified in such common model organisms as *Drosophila melanogaster*, *Caenorhabditis elegans* and *Saccharomyces cerevisiae*.

## 2. Activities and Fidelity of PrimPol

Since 2013, the biochemical activities of human PrimPol have been extensively studied. In vitro, human PrimPol possesses properties of a translesion DNA polymerase. Like other translesion DNA polymerases, PrimPol lacks 3′–5′-exonuclease activity and exhibits low fidelity of DNA synthesis. In the presence of Mg^2+^ ions as a cofactor of DNA polymerization, PrimPol makes one error per 10^2^–10^5^ nucleotides on undamaged DNA templates [30,31,32,33], an error rate comparative with the fidelity of the error-prone Y-family, human Pol η, Pol ι and Pol κ [13]. However, the error specificity of PrimPol uniquely differs from other human DNA polymerases. In particular, PrimPol has a preference to generate base insertions and deletions (indels) over base misincorporations [30,34]. The high rate of indel mutations potentially leads to frame-shift mutagenesis and has a deleterious effect on cells. PrimPol also preferentially incorporates non-complementary nucleotides opposite the templating bases C and G and efficiently extends from primers with terminal mismatched base pairs contributing to mutation fixation [30]. In addition to low fidelity, another feature that PrimPol shares with translesion DNA polymerases is low processivity of DNA synthesis, as it incorporates only a few nucleotides per binding event [35]. The poor processivity can be explained by the lack of contacts with DNA (see below) and the low affinity of PrimPol to DNA [33,36].

It was shown that both DNA polymerase and DNA primase activities of PrimPol are significantly stimulated by Mn^2+^ ions [27,31,33]. The stimulation of the catalytic activity can be explained by the strong increase of PrimPol’s affinity to DNA in the presence of Mn^2+^ ions [31,32,33]. However, Mn^2+^ ions decrease the fidelity of nucleotide incorporation by PrimPol [31,32,33]. Previous studies demonstrated that Mn^2+^ ions alter catalytic properties of many translesion DNA polymerases and, in particular, stimulate catalysis and DNA damage bypass by human Pol ι [37,38], Pol λ [39] and Pol μ [40]. Mn^2+^-dependent DNA synthesis may therefore play a role in the regulation of TLS in vivo. However, the direct evidence of the requirement of Mn^2+^ ions as a physiological cofactor for translesion DNA polymerases is lacking.

As previously mentioned, human PrimPol possesses properties of a translesion DNA polymerase in vitro, and bypasses several types of DNA lesions [26,27,33,41] (Table 1). PrimPol perform efficient and quite accurate synthesis through the most common type of oxidative damage—8-oxo-G (Table 1). Interestingly, Keen B.A. et al. observed the efficient and error-free bypass of *cis-syn* cyclobutane pyrimidine dimers by the PrimPol catalytic core (residues 1–354), but did not observe bypass by full-length PrimPol protein [35]. These data suggest that the TLS activity of PrimPol may be modulated by conformational changes.

Contrasting results were obtained on DNA templates with an abasic site (AP-site). Several studies did not observe AP-site bypass by PrimPol [26,42], whereas in the study by Garcia-Gomez S. et al., PrimPol efficiently bypassed an AP-site by the lesion “skipping” mechanism (also called pseudo-TLS and the template “scrunching” mechanism) [27] (Table 1). In this latter case, PrimPol does not insert a nucleotide opposite the AP site but skips the lesion, copying the next template base available. This scenario suggests that PrimPol re-anneals the primer to the nucleotide located downstream of the lesion and loops out the templating lesion. A similar mechanism was reported for T–T (6-4) photoproducts by Mouron S. and Martinez-Jimenez M. et al. [34,41]. It is likely that this mechanism is sequence-dependent and is facilitated by flanking microhomologies [27,34,41]. The preference of PrimPol to generate base deletions on undamaged DNA is in agreement with the observation of the lesion skipping mechanism. Along with different sequence context, the differences in reaction conditions could contribute to inconsistency in TLS activities of PrimPol in experiments of different groups.

PrimPol also possesses an unique primase activity and is the second primase found in human cells after the Pol α-primase complex. Strikingly, unlike the Pol α-primase, PrimPol can catalyze the incorporation of deoxyribonucleotides (dNTPs) to make primers during *de novo* DNA synthesis [26,27]. The incorporation of dNTPs into the newly synthesized DNA does not require the removal of an RNA primer after the initiation of DNA synthesis.

The primase activity of PrimPol is dependent on a template T and on ATP or dATP as a starting nucleotide. On homopolymeric single-stranded DNA, PrimPol possesses primase activity only on a poly(dT) template [26]. Accordingly, on a 3′-GTCC-5′ template PrimPol preferentially forms initiating dinucleotides 5′-A-dG-3′, and 5′-dA-dG-3′ PrimPol incorporates deoxyribo- and ribonucleotides with similar efficiency for initiation of *de novo* synthesis but prefers deoxyribonucleotides at the second position [27]. In contrast, the initiation of the primer synthesis by the eukaryotic Pol α-primase complex requires ATP or GTP on poly(dT) and poly(dC) templates, with a slight preference for an ATP substrate [43,44,45]. Interestingly, PrimPol possesses higher affinity towards poly(dT) as well as towards poly(dG) and G-quadruplexes [46]. The template specificity during DNA binding and initiation of synthesis may reflect a dependence of PrimPol on certain natural initiation sites and likely plays a role in the regulation of PrimPol repriming activity in vivo. Indeed, it was shown that PrimPol initiates specific repriming almost immediately downstream of the G-quadruplex structures in vitro [46].

Finally, PrimPol has been reported to be able to connect two separate oligonucleotides by an oligonucleotide end bridging mechanism. To this end, PrimPol was shown to be able to induce alignment of non-complementary oligonucleotides based on microhomologies as short as one or two base pairs, and use these aligned oligonucleotides as a template for elongating the opposite strand. Such activity would open up the possibility of a role for PrimPol in non-homologous end joining [34]. Moreover, it was suggested that PrimPol possesses the terminal transferase activity (template-independent primer extension activity) which is stimulated by Mn^2+^ ions. In this case, PrimPol extends a primer to a homopolymeric strand, which is non-complementary to the template strand [35]. However, a conventional terminal transferase activity was not observed by Martínez-Jiménez M. et al. Alternatively, it was suggested that the transferase activity of PrimPol can be a result of the connecting activity. In this scenario, PrimPol extends DNA oligonucleotides incorporating the sequence homologous to the connected nucleotide [34]. Future studies are required to determine the role of methodological differences which may affect the activities of PrimPol in vitro, verify the terminal transferase and the connecting activities of PrimPol in vitro and in vivo and elucidate their biological roles in cells.

## 3. Structure of PrimPol

**Figure 1:**
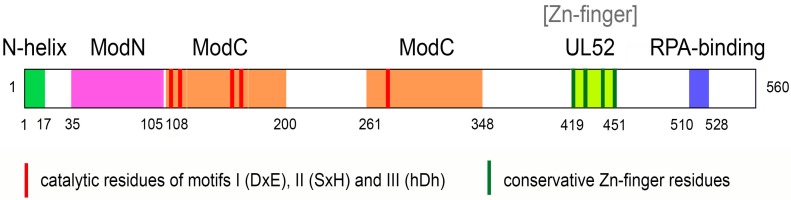
The schematic domain structure of human PrimPol. The N-helix, the Module N (ModN) and Module C (ModC) modules, the C-terminal zinc finger (ZnF) and replication protein A (RPA)-binding domains as well as conservative catalytic residues of I, II and III-motifs and Cys residues coordinating [Zn] are indicated.

Human PrimPol is a 560 amino acid protein. It contains an N-terminal AEP-like catalytic domain and a C-terminal zinc finger (ZnF) domain that forms contacts to the DNA template [25,27,35]. The conserved I, II, III-motifs in the AEP-like domain are required for both the DNA polymerase and primase activities (Figure 1) [26,27].

The first crystal structure of the N-terminal catalytic core of human PrimPol (residues 1–354) in a ternary complex with a DNA template-primer, an incoming dATP and one Ca^2+^ ion was recently reported [36]. A comparison of human PrimPol with catalytic cores of other DNA polymerases and PriS provides some insight into the ability of PrimPol to function as both a polymerase and a primase.

Generally, the structures of most *DNA polymerases* resemble a *right hand. Y-family translesion* DNA polymerases contain the palm, fingers, thumb and little finger (or polymerase-associated domain), wherein the palm domain contains the active site, the finger domain interacts with the nascent base pair, the little finger and the thumb domains contact with the primer and the templating DNA [47]. However, the PrimPol catalytic core contains only the N-helix (an N-terminal helix, residues 1–17) and two modules called Module N (ModN) (residues 35 to 105) and Module C (ModC) (residues 108 to 200 and 261 to 348). The N-helix is connected to the ModN via a long flexible linker (residues 18–34) [36].

The ModC module encompasses functions of both the finger and palm domains and harbors key active site residues interacting with the nascent T-dATP base pair. The conserved ModC motifs I (DxE) and III (hDh) contain the acidic catalytic residues Asp114/Glu116 and Asp280, which are involved in coordination of Mе^2+^ ions, while motif II (SxH) contains S167 and His169 participating in the incoming nucleotide binding. Mutations in these residues abrogate the polymerase and primase activities of human PrimPol [26,27,28,35]. Residues 201 to 260 inside ModC correspond to an unstructured region, which may have a regulatory role.

In PrimPol, ModN together with ModC functions as the finger domain and these modules are in contact with the template DNA strand and the templating base (T). The N-helix interacts with the template strand and resembles the little finger domain in Y-family polymerases but makes far fewer contacts in the major groove. Moreover, PrimPol does not have an analogue to the thumb domain to grip the template-primer. As a result, PrimPol demonstrates an almost complete lack of contacts to the DNA primer strand. This feature can play a key role in the primase activity of PrimPol, as the lack of contacts to the DNA primer strand eliminates the need for a pre-existing primer and leaves room for a dNTP at the initiation site during *de novo* DNA synthesis. The PrimPol catalytic core structure also differs from the structure of the primase subunit PriS because PriS has no equivalent of the N-helix. The contacts of the N-helix with the template DNA, likely, play an important role in the DNA polymerase activity of PrimPol [36].

The C-terminal domain of PrimPol contains a conserved ZnF motif (consisting of key residues C419, H426, C446, C451), which shares high sequence similarity with the viral UL52 primase domain. The Zn^2+^ ion is coordinated by the first conserved cysteine and histidine residues of the motif [26,35,41]. The presence of Zn^2+^ ions in a protein sample of the PrimPol C-terminal domain was confirmed by inductively coupled mass spectrometry [35]. The structure of the C-terminal domain of PrimPol has not been reported, but the C-terminal ZnF is indispensable for *de novo* synthesis by PrimPol while not being necessary for primer elongation [28,35]. In particular, mutations of residues C419 and H426 abrogate the primase activity of PrimPol but retain its DNA polymerase activity [28,35,41]. Nevertheless, the deletion and mutations of the ZnF modulate the processivity and fidelity of DNA synthesis by PrimPol as the presence of the ZnF reduces the processivity of the enzyme and allows a slower, higher-fidelity incorporation of complementary nucleotides [35]. The mutation of the ZnF also abrogates template-independent dNTP incorporation by PrimPol in the presence of Mn^2+^ ions [35].

The C-terminal domain is required for the binding to single-stranded DNA downstream of the primer-template junction [35] and likely is responsible for template recognition during repriming. It was also suggested that the ZnF may function as a “translocation” site to capture the initiating nucleotide triphosphate [36] analogous to the Fe–S domain in the PriS regulatory subunit PriL [21]. Moreover, the C-terminal domain was shown to be involved in protein interactions with replication protein A (RPA) (see below) [28,30,48]. Altogether, the data suggest that the C-terminal domain is a key regulator of PrimPol function.

The structures of PrimPol with DNA lesions are yet to be determined. Human PrimPol replicates through photoproducts including the highly distorting T–T (6-4) lesion but, in contrast with Pol η, PrimPol has a constrained active-site cleft with respect to the templating base [36]. This suggests that PrimPol bypasses photoproducts and other bulky DNA lesions by looping the lesion out in the space between the ModN and the N-helix near the flexible linker and also explains the skipping of DNA lesions observed in some works [27,34,41].

## 4. Functions of PrimPol in Cells

### 4.1. The Role of PrimPol in Nuclear Replication and DNA Translesion Synthesis

Similar with other AEP enzymes, PrimPol possesses the versatile activities which provide a possibility to participate in different cellular processes including replication in unperturbed cells, DNA damage tolerance and, possibly, repair [24]. Studies demonstrated that PrimPol plays an important role in maintaining genome stability by protecting cells from replication stress derived from DNA damage, as well as by assisting fork progression on undamaged DNA. In accordance with these roles, PrimPol was shown to be recruited to the sites of DNA damage and stalled replication forks in the nucleus in vivo [26,28,41].

Mammalian and avian cells deficient in PrimPol display sensitivity to ultraviolet (UV) irradiation [26,28,35,41,49,50,51], indicating that PrimPol is important for recovery from UV damage. Because the loss of PrimPol in human xeroderma pigmentosum variant (XPV) cells lead to an increase in UV sensitivity, PrimPol’s contribution to tolerance of UV photoproducts is likely to involve a pathway that is independent of Pol η [26]. Furthermore, chicken *PRIMPOL^−/−^* DT40 cells are also hypersensitive to cisplatin and methylmethane sulfonate; this effect was not epistatic to the Pol η- and Pol ζ-dependent pathways [52]. Finally, PrimPol appears not to play a major role in recovery from double-strand breaks as human *PRIMPOL^−/−^* cells showed little or no hypersensitivity to ionizing radiation [28].

Moreover, PrimPol is crucial for recovery of stalled replication forks in HeLa and DT40 cells after treatment with the dNTP depleting agent hydroxyurea and chain-terminating nucleoside analogues [28,41,52], and possibly plays an important role in replication even in unperturbed cells. In the absence of induced DNA damage, disruption of PrimPol function in mammalian cells slows down replication and induces replicative stress, thereby leading to the accumulation of DNA breaks and chromosome instability [26,41]. In particular, PrimPol may facilitate the replication across non-B DNA [46]. However, L. Wan et al. and B. Pilzecker et al. observed only modest or no effect of PrimPol defects on the replication speed during unperturbed replication in human cells [28,51].

PrimPol may restart a stalled replication fork by acting as either a translesion DNA polymerase or by repriming DNA synthesis downstream of the lesion. Biochemical studies have indicated that PrimPol is capable of TLS synthesis in vitro. However, recent in vivo studies with a zinc-finger (ZnF) primase-null PrimPol mutant in human and avian DT40 cells (see below) suggested that the primary function of PrimPol in nuclear replication is repriming at sites of DNA damage and at stalled replication forks on the leading strand. It was shown that PrimPol reprimes efficiently downstream of UV-induced DNA lesions, AP-sites and cisplatin lesions [35,41,51,52]. Moreover, it was suggested in Rad51-depleted and UV-treated cells PrimPol promotes dysregulated excessive elongation of nascent DNA by repriming after UV-induced lesions accumulated behind the replication fork. Rad51 recombinase protects the DNA synthesized before UV irradiation from degradation and prevents PrimPol-mediated repriming and excessive elongation of nascent DNA after UV irradiation [53].

It is likely that the primase activity of PrimPol also plays a pivotal role in the re-initiation of DNA synthesis after dNTP depletion by hydroxyurea and chain termination with nucleoside analogues [41,52]. It was also suggested that PrimPol contributes to replication across G-quadruplexes using close-coupled downstream repriming mechanisms on the leading DNA strand [46]. Taken together, the most likely role of PrimPol is to initiate *de novo* DNA synthesis downstream of not only DNA lesions, but also non-B DNA structures during normal chromosomal duplication.

The discovery of the repriming role of PrimPol in cells explains the previous evidence for chromosomal single-stranded gaps formed on the leading strand in S phase during replication and gap-filling during TLS in G2 phase of the cell cycle [54,55]. The mechanism of repriming is especially important on the leading strand which is replicated continuously. The remaining gaps can be filled by translesion DNA polymerases or restored by homology-directed repair. More information about repriming function of PrimPol in cells can be found in recent review [56].

However, a TLS function of PrimPol in cells cannot be completely ruled out. First, it is possible that the PrimPol ZnF mutants used as primase-deficient but DNA polymerase-proficient variants in the separation of function studies lack some posttranslational modifications or interactions with accessory proteins. Mutations of the ZnF may also affect PrimPol stability and folding. Any of these scenarios could impair the activity of the ZnF mutants and hence lead to an interpretation that overestimates the role of the primase activity. Second, several studies have evidenced a role for the polymerase activity of PrimPol in replication. In particular, Keen B.A. with co-authors suggested that in avian *PRIMPOL*^−/−^ DT40 cells expressing human PrimPol, its primase activity is required to restore wild-type replication fork rates after UV irradiation, while the DNA polymerase activity of PrimPol is sufficient to maintain regular replisome progression in unperturbed cells [35]. Moreover, the African trypanosome *Trypanosoma brucei* encodes two forms of PrimPol-like proteins, PPL1 and PPL2. Both PPL1 and PPL2 proteins are translesion DNA polymerases and only the PPL1 form possesses primase activity. However, in the bloodstream form of trypanosome, PPL2 was suggested to be essential for the post-replication tolerance of DNA damage using its TLS activity, while PPL1 appears to be dispensable [29]. PPL2 was also required to complete genome replication in *T. brucei* even in the absence of external DNA damage. Therefore, it is possible that functions of PrimPol-related proteins and the regulation of their activity differ among species.

### 4.2. Functions of PrimPol in Mitochondria

As previously mentioned, a considerable fraction of PrimPol in cultured human cells localizes to mitochondria [27], whereby it may be available to assist Pol γ, the mitochondrial replicase, in the synthesis of mitochondrial DNA (mtDNA). In support of a mitochondrial function of PrimPol, its silencing in HEK293T cells causes a decrease in mtDNA copy number [27], and human or mouse cells lacking PrimPol show delayed recovery after transient drug-induced mtDNA depletion [27]. However, as *PRIMPOL^−/−^* knockout mice are viable, PrimPol is not essential for mtDNA maintenance [27].

A role for PrimPol in mtDNA maintenance is further supported by the fact that the DNA polymerization activity of PrimPol is affected by bona fide components of the mitochondrial replisome. Specifically, PrimPol interacts with and is inhibited by the mitochondrial single-stranded DNA-binding protein (mtSSB), most likely due to displacement of PrimPol from single-stranded DNA (ssDNA) [30]. Furthermore, PrimPol is stimulated by the mitochondrial replicative helicase Twinkle [42] and by polymerase delta-interacting protein 2 (PolDIP2) (Table 2) [50], a protein initially discovered as a Pol δ interactor [57] and that at least in some cell types appears to be localized to the mitochondrial matrix where also PrimPol is found [27,58].

Despite these functional interactions, the precise contribution of PrimPol to mtDNA maintenance has remained unclear. Intuitively, given its translesion synthesis abilities, PrimPol could be expected to synthesize past oxidative lesions created due to the high levels of reactive oxygen species that originate from the mitochondrial electron transport chain. However, PrimPol is unable to assist the mitochondrial replisome in bypassing the most common types of oxidative lesions, 8-oxo-G and abasic sites, both of which pose considerable blocks to the progression of the mitochondrial replication machinery [42]. Therefore, as has been suggested for the nucleus, the main contribution of PrimPol to mtDNA maintenance is likely to involve its ability to reprime replication. Indeed, our current unpublished data suggest PrimPol to be required for the repriming of stalled mtDNA replication after UV damage or treatment with the chain terminating nucleoside analog ddC in mouse cells, and to be able to provide primers for DNA replication by Pol γ both in vivo and in vitro [59].

Given that human mtDNA contains a number of sites that can form G-quadruplex structures [60,61,62,63,64], the repriming of mitochondrial replication may be required even in the absence of mtDNA damage. Because PrimPol has the ability to reprime replication immediately downstream of G4 structures in vitro [46], it may facilitate replication progression past these, and possibly other, mtDNA secondary structures. Depletion and/or deletions of mtDNA are implicated in rare genetic mitochondrial disorders, and causative gene defects include mutations in Pol γ, the Twinkle helicase and other factors involved in mtDNA maintenance [5,6]. The involvement of PrimPol in proper maintenance of mtDNA therefore opens up the possibility that PrimPol defects could give rise to mitochondrial pathologies.

## 5. Regulation of PrimPol Activity in Cells

To date, little is known about the mechanisms that regulate TLS and the re-initiation of DNA synthesis by PrimPol at the sites of DNA damage. Generally, translesion DNA polymerases are tightly regulated in the cell due to their high mutagenic potential. Numerous protein factors control the catalytic activity of DNA polymerases and their access to the replication fork. In nuclei, RPA and the trimeric protein-clamp proliferating cell nuclear antigen (PCNA) play a key role in the regulation of DNA polymerase activity. In mitochondria, mtSSB and helicase Twinkle are essential for DNA replication by Pol γ.

PCNA is involved in the replication process as a processivity factor. Functional interaction with PCNA facilitates access to replication fork and stimulates activity of both replicative and translesion DNA polymerases [65,66,67,68]. However, PrimPol does not interact with PCNA in vivo and is not stimulated by PCNA in vitro, suggesting that the regulation of PrimPol differs from other DNA polymerases and is independent of PCNA [30].

The main functions of RPA and mtSSB include stabilization of single-stranded DNA and positioning of proteins for the formation of DNA-protein and protein-protein complexes. RPA and mtSSB interact with PrimPol, and RPA has been shown to regulate PrimPol localization in cells in response to DNA damage and replication stress in vivo (Table 2) [28,30,48]. In particular, the deletion of the C-terminal RPA binding domain of PrimPol abrogates its interaction with RPA and nuclear foci formation after treatment of cells with *hydroxyurea*, i*onizing radiation* and UV irradiation [28,48]. Therefore, it is likely that RPA plays a key role in the recruitment of PrimPol to the stalled replication fork, and that mtSSB may play a similar role in mitochondria. Interestingly, while both RPA and mtSSB inhibit the DNA polymerase and primase activities of PrimPol on short ssDNA templates in vitro [30,42,69], RPA stimulates PrimPol activity on long M13 ssDNA templates [48,69]. The biochemical results are consistent with a model where full coating of ssDNA by RPA, such as is expected during normal replication, prevents PrimPol from accessing the DNA. In contrast, under conditions where the ssDNA is not fully coated with RPA and simultaneous binding of RPA and PrimPol is possible, RPA stimulates priming and polymerization by PrimPol. The latter scenario has been suggested to take place during replicative stress when the leading strand polymerase and the replicative helicase become uncoupled and longer stretches of ssDNA are exposed. The interaction between RPA and PrimPol is mediated by two acidic motifs in the C-terminal RPA binding domain (RBD) of PrimPol that have the ability to bind the basic cleft on the N-terminus of the RPA1 subunit in vitro [30,48]. However, in vivo the first of these RPA-binding motifs (amino acids 510–528 of PrimPol) has been suggested to be the primary mediator of the PrimPol-RPA interaction, and it is required for chromatin recruitment of PrimPol following UV irradiation [48].

Recently, it was found that the DNA polymerase activity of PrimPol is stimulated by the mitochondrial helicase Twinkle (Table 2) [42]. *Twinkle stimulates synthesis of longer DNA replication products by PrimPol at high dNTP concentration. Interestingly, the helicase activity of Twinkle is not required for this stimulation. The stimulation by Twinkle was also not specific to damaged DNA as it was observed on undamaged DNA.*

Recently it was also shown that the DNA polymerase activity of human PrimPol is stimulated by interaction of its AEP domain with PolDIP2 (Table 2) [50]. PolDIP2 enhances the binding of PrimPol to DNA and stimulates the processivity of DNA synthesis but does not stimulate the primase activity of PrimPol [50]. The depletion of PolDIP2 in UV irradiated human cells causes a decrease in the replication fork rate, similar to that observed in *PrimPol^−/−^* cells. Moreover, no further decrease in replication rate was observed when PolDIP2 was depleted in *PrimPol^−/−^* cells. These data suggest that PrimPol and PolDIP2 work epistatically in the same *pathway to promote DNA replication in the presence of UV damage* [50].

Importantly, PolDIP2 also interacts with PCNA, the regulatory p50 subunit of replicative Pol δ and several translesion DNA polymerases [57,70,71]. It was shown that PolDIP2 stimulates Pol δ by increasing its affinity for PCNA binding [70]. Therefore, PolDIP2 may play a role in the coordination of replisome proteins providing docking sites for DNA polymerases and PCNA and facilitating their functional interactions. However, it was shown that PolDIP2 together with PCNA inhibits the DNA polymerase activity of PrimPol in vitro. These data argue against the “docking hypothesis” and suggests that PolDIP2 does not play a “bridging role” between PrimPol and PCNA [50].

PolDIP2 has been found both in the nucleus and mitochondria. While Klaile et al. showed that endogenous human and rat PolDIP2 localized primarily in the cytoplasmic and nuclear fractions, with lesser amounts entering the mitochondria [72], Cheng X. et al. and Xie B. et al. localized PolDIP2 almost exclusively in mitochondria [58,73]. Furthermore, it was shown that PolDIP2 is associated with the mitochondrial DNA nucleoid and it co-immunoprecipitated with mtSSB and mitochondrial transcription factor A (TFAM) [58]. Nevertheless, the role of PolDIP2 as a regulator of PrimPol function in mitochondria has been questioned by biochemical studies, because *the interaction between PolDIP2 and PrimPol is mediated by the N-terminal region of PolDIP2 that also contains the mitochondrial targeting signal that is likely cleaved off upon mitochondrial entry* [50]. The truncated form of PolDIP2 lacking the first 50 amino acids did not stimulate the DNA polymerase activity of PrimPol [50]. Therefore, it is possible that the stimulatory effects of PolDIP2 on PrimPol may be rather nuclear than mitochondrial.

Finally, to date, no proteins that act to stimulate the primase activity of PrimPol have been reported excepting for RPA [48,68]. Therefore, the mechanisms regulating repriming by PrimPol remain to be elucidated.

## 6. PrimPol Dysfunction and Disease

*PRIMPOL^−/−^* knockout mice are viable but *PRIMPOL^−/−^* deficient cells show replication stress, genetic instability and defects in mitochondrial replication [27]. It is likely that PrimPol defects can be compensated in vivo by alternative mechanisms such as the use of (other) translesion DNA polymerases and helicases to bypass DNA damage and non-B structures, template switching mechanisms, homology-dependent repair and firing of dormant origins. Indeed, it was shown that avian DT40 cells deficient only in PrimPol or in Pol η/Pol ζ are viable and proliferate with nearly normal kinetics. However, cells deficient in PrimPol and Pol η/Pol ζ proliferated slowly and exhibited increased cell death. These data suggest that repriming by PrimPol and TLS by Pol η/Pol ζ are compensatory DNA damage tolerance mechanisms [52].

Nevertheless, the established roles of PrimPol in DNA damage tolerance and mitochondrial DNA replication suggest that PrimPol mutations could lead to some inherited diseases including cancer predisposition and mitochondriopathies. To date, however, little is known about the influence of PrimPol defects on human health. Recent analysis of gene expression data from The Cancer Genome Atlas (TCGA) demonstrated anti-mutagenic activity of PrimPol in genome maintenance and suggested a possible protective role of PrimPol in human breast cancer [51]. The study reported a high number of *PRIMPOL*-deficient tumors in breast cancer patients diagnosed with invasive lobular and ductal carcinoma, and the *PRIMPOL^−/−^* tumors were found to exhibit a mutation load that was nearly twice as high as in *PRIMPOL*-proficient tumors.

Only one missense *PRIMPOL* mutation, a naturally occurring minor PrimPol variant, has been reported to potentially associate with human disease [74]. This missense mutation (NM_152683.2: c.265T > G) results in the Y89D amino acid change in PrimPol and causes global alterations in polymerase domain structure and significantly decreased PrimPol affinity for both dNTPs and DNA. Being unable to interact firmly with DNA, the PrimPol^Y89D^ variant has dramatically lower processivity than wild-type PrimPol, which in turn causes a significant slowing of replication fork progression and increased UV-sensitivity in vivo [75]. Zhao et al. [74] hypothesized a link between the PrimPol^Y89D^ variant and the human ocular disease high myopia. This mutation was found in a single family and four additional sporadic patients with high myopia. Within the family, the mutation is inherited in an incomplete autosomal dominant manner, and when heterozygous, a lighter form of the disease results [74]. Nevertheless, further screening of genomes did not confirm this hypothesis as the PrimPol^Y89D^ variant was found among individuals with high myopia, other forms of genetic eye diseases and normal controls [76].

Many translesion DNA polymerases promote the resistance of cancer cells to chemotherapy [77,78]. As PrimPol is involved in tolerance to DNA damage, it can be assumed that PrimPol may play a role in the development of tolerance to some chemotherapeutic drugs inhibiting DNA replication and may therefore represent a promising drug target for the treatment of chemotherapy-resistant tumors.

## 7. Conclusions

Many human translesion DNA polymerases have been found and characterized in the last 15 years, and PrimPol is the most recent DNA polymerase to be identified. PrimPol is unique due to its dual activities as a translesion DNA polymerase and a primase. These activities provide extreme flexibility of DNA damage bypass as PrimPol can contribute to bypass through classical TLS, by skipping the lesion and/or by *de novo* priming of DNA synthesis downstream of the lesion. Since its discovery in 2013, extensive biochemical and structural studies have provided insight into the biochemical properties and functions of PrimPol and the mechanisms of its DNA polymerase and primase activities. Growing evidence suggests that the main biological function of human PrimPol during replication of chromosomal DNA is a repriming of stalled replication downstream of DNA damage or naturally occurring obstacles. However, the mechanisms that regulate the repriming by PrimPol in cells are yet to be understood. The functions of PrimPol in mitochondria and the association of PrimPol defects with human diseases are also largely unexplored and constitute important directions for future research.

## Figures and Tables

**Table 1:** The translesion synthesis (TLS) activity of human PrimPol.

DNA Damage		PrimPol TLS In Vitro
oxidative lesions	8-oxo-G	- bypasses 8-oxo-G incorporating dATP and dCTP with equal efficiency [26,27]; - bypasses 8-oxo-G and preferentially incorporates dC [33,42]
	TG (thymidine glycol)	- does not bypass TG [26,35]; - PrimPol _1–354_ incorporates a nucleotide opposite TG but cannot extend from the lesion [35]
photo-products	*cis-syn* T–T dimers	- bypasses CPD *cis-syn* T–T dimers [41]; - does not bypass *cis*-syn T–T dimers but extends a primer terminus with two dA residues annealed opposite the T–T CPD [26]; - PrimPol_1-354_ bypasses *cis-syn* T–T dimers with high efficiency and fidelity [35]
	T–T (6–4) photoproducts	- bypasses T–T (6–4) photoproducts in error-prone manner incorporating dTTP opposite 3′Т and dGTP/dCTP opposite 5′Т [26] or by skipping mechanism [27,34,41]
abasic sites		- does not bypass lesion [26,35,42]; - bypasses lesion with high efficiency using skipping mechanism [27,34]; - bypasses with very weak efficiency and shows nearly equal preference for either skipping the abasic site or inserting dAMP [33]
deoxyuracil		- bypasses as T and incorporates dATP opposite the lesion [35]

**Table 2:** Proteins interacting with PrimPol.

▪ Protein-Partner	Localization of Protein in DNA Compartment	Effect on PrimPol Activity
**▪** RPA (replication protein A)	nuclear	inhibits primase and polymerase activities on short DNA templates [30,42,69] but stimulates primase and polymerase activities on long DNA templates when non-saturating in vitro [48,69], targets PrimPol to DNA damage sites in nuclei in vivo [28,30,48]
**▪** mtSSB (mitochondrial single-stranded DNA-binding protein)	mitochondrial	inhibits primase and polymerase activities on short DNA templates in vitro [42]
**▪** PolDIP2 (polymerase delta-interacting protein 2)	mitochondrial and possibly nuclear	stimulates DNA polymerase activity in vitro [50]
**▪** Twinkle	mitochondrial	stimulates DNA polymerase activity in vitro [42]
